# Supplementary material for: GCACL-Rec: A study on conversational recommendation via global context-aware and multi-view contrastive adversarial joint learning
Source: PLoS One. 2025 Oct 30;20(10):e0335176. doi: 10.1371/journal.pone.0335176 (PMC12574935; doi:10.1371/journal.pone.0335176)
Supplement: Attachment — Submitted filename: Response to Reviewers.docx [file pone.0335176.s001.docx]

Response Letter

GCACL-Rec: A Study on Conversational Recommendation via Global Context-Aware and Multi-View Contrastive

Adversarial Joint Learning

Xianghui Li,Xiaowen Liu,Xinhuan Chen and Ming Ma

**Editor:**

1. *Please ensure that your manuscript meets PLOS ONE's style requirements, including those for file naming. The PLOS ONE style templates can be found at:*

**RE:** Thank you for bringing the formatting issues to our attention. We have thoroughly reviewed the formatting to ensure it is correct.

2. *Please note that PLOS One has specific guidelines on code sharing for submissions in which author-generated code underpins the findings in the manuscript. In these cases, we expect all author-generated code to be made available without restrictions upon publication of the work. Please review our guidelines at [https://journals.plos.org/plosone/s/materials-and-software-sharing#loc-sharing-code](https://journals.plos.org/plosone/s/materials-and-software-sharing#loc-sharing-code) and ensure that your code is shared in a way that follows best practice and facilitates reproducibility and reuse.*

**RE:** Thank you for your reminder regarding the code sharing guidelines. As the code is still under active development and will serve as the basis for our subsequent research, we would prefer not to make it publicly available at this stage. To address this, we have added a statement in the conclusion of the manuscript noting that interested readers may contact the corresponding author, and access can be provided upon reasonable request and evaluation. We hope this arrangement will be acceptable, and we sincerely appreciate your understanding.

3. *We note that the grant information you provided in the ‘Funding Information’ and ‘Financial Disclosure’ sections do not match. When you resubmit, please ensure that you provide the correct grant numbers for the awards you received for your study in the ‘Funding Information’ section.*

**RE:** We sincerely apologize for the inconsistencies in the “Funding Information” and “Financial Disclosure” sections of our previous submission. This was due to an oversight in entering the grant number. After review, we have corrected this information and incorporated it into the cover letter, revised as follows:

This research was supported by the 2024 Graduate Student Innovation Program of Beihua University (Project No. 2024058). The funding provided support for data collection, basic research activities, and partial dissemination of academic findings. The funders had no role in study design, data collection and analysis, decision to publish, or preparation of the manuscript. There was no additional external funding received for this study.

4. *PLOS requires an ORCID iD for the corresponding author in Editorial Manager on papers submitted after December 6th, 2016. Please ensure that you have an ORCID iD and that it is validated in Editorial Manager. To do this, go to ‘Update my Information’ (in the upper left-hand corner of the main menu), and click on the Fetch/Validate link next to the ORCID field. This will take you to the ORCID site and allow you to create a new iD or authenticate a pre-existing iD in Editorial Manager.*

**RE:** Thank you for the reminder regarding the ORCID iD requirement. We confirm that ORCID iDs have been provided for all authors and properly linked in the submission system.

5. *If the reviewer comments include a recommendation to cite specific previously published works, please review and evaluate these publications to determine whether they are relevant and should be cited. There is no requirement to cite these works unless the editor has indicated otherwise.*

**RE:** Thank you for your guidance regarding the reviewer’s recommendation. We have carefully reviewed the suggested publications and found them to be relevant and valuable for enriching the related knowledge in the graph domain. Accordingly, we have incorporated and properly cited these works in the revised manuscript.

**Reviewer :1**

*1.The proposed GCACL-Rec model is ambitious and multi-component, but the overall model description would benefit from a clearer visual pipeline or algorithm box that concisely summarizes each stage (MSGNN, MPACL, NDF). This would help readers track the execution flow.*

**RE:** We thank the reviewer for the constructive suggestion. We agree that a clearer summary of the GCACL-Rec pipeline would help readers better follow the multi-component design (MSGNN, MPACL, and NDF). To address this, we have added a dedicated algorithm box in the revised manuscript (see Algorithm 1: Training Process of GCACL-Rec). The algorithm concisely presents the end-to-end execution flow, highlighting the key modules — graph modeling (MSGNN), representation learning, multi-perspective contrastive & adversarial learning (MPACL), and hybrid prediction (NDF). This complements the detailed textual description and serves as a visual guideline for readers to quickly grasp the entire workflow. [The specific modifications are highlighted in yellow on page 13 of the revised draft:](D:\\论文-PPT\\论文\\plos one\\ESP\\Marked-up copy of the manuscript.pdf" \l "page=13)

Additionally, We have revised the manuscript to more clearly articulate the rationale for using the Neural Decision Forest (NDF) in our research. Traditional session recommenders typically adopt an encoder–predictor paradigm where the predictor is a linear dot-product/softmax layer. While simple, this predictor has limited capacity to capture non-linear and multi-modal next-item intent boundaries, particularly under the noisy clicks and random behaviors that are common in session data. As a result, even strong encoders can be bottlenecked by the low-capacity predictor. To address this, recent work[21] demonstrated that augmenting the predictor with a high-capacity tree-inspired module, together with a random-behavior alleviator and capacity control (pruning), yields consistent performance gains across different backbones. Following this perspective, we replace the weak linear head in our framework with a Neural Decision Forest (NDF) predictor. The NDF branch applies James–Stein shrinkage to denoise the latent session vector and then uses an ensemble of differentiable trees to model more complex, non-linear decision boundaries. A merger mechanism fuses the NDF with the softmax branch, balancing interpretability and robustness. [The specific modifications are highlighted in yellow on page 11 of the revised draft:](D:\\论文-PPT\\论文\\plos one\\ESP\\Marked-up copy of the manuscript.pdf" \l "page=11)

However, Most session recommenders use an encoder–predictor setup: the encoder turns a session into a vector, and a predictor scores items. A common linear dot-product predictor has low capacity and struggles with noisy or random clicks, bottlenecking even strong encoders. Recent work demonstrates that incorporating a high-capacity, tree-inspired module—alongside denoising the session vector and pruning to manage capacity—consistently improves accuracy. Following this, we utilize a global, context-aware encoder and replace the linear head with a Neural Decision Forest (NDF) that incorporates denoising, allowing the final decision boundary to better separate true next-item intent from noise. The concatenated feature from h_u and h_s is first processed with James-Stein shrinkage to reduce variance from small sample sizes:

*2.Terms like “supernodes”, “hypernodes”, and “global graph” are used interchangeably. Standardizing this terminology and releasing training scripts or code snippets would significantly improve reproducibility.、*

**RE:** Thank you for your valuable comments on my manuscript. Regarding the confusion you mentioned about terms such as “supernode,”“hypernodes,” and “global graph”，I have reviewed the paper and made relevant revisions, uniformly changing “super node” to “hyper node” and adding corresponding annotations in the paper regarding the use of hyper nodes and explanations of global graphs. For revisions made to the paper, please refer to the annotated paragraphs in the revised draft, specifically the global graph neural network module in the methods section. [The specific modifications are highlighted in yellow on page 7 of the revised draft:](D:\\论文-PPT\\论文\\plos one\\ESP\\Marked-up copy of the manuscript.pdf" \l "page=7)

In this paper, we use the term hypernodes consistently to denote auxiliary nodes that aggregate structurally or semantically similar sessions.Similarly, we use global graph to describe the integrated cross-session structure.

*3.Several figures in the manuscript (e.g., Fig. 1: overall model architecture, Fig. 2–5: ablation and comparison results) appear blurry and lack sufficient resolution when viewed in print or on screen. This significantly affects the readability of key visual elements such as graph legends, axis labels, and numerical values. The authors are strongly encouraged to regenerate all figures with higher resolution (preferably vector graphics such as PDF, SVG, or EPS formats) to ensure visual clarity and professional presentation quality.*

**RE:** We appreciate the reviewer's important point. In the revised manuscript, we have regenerated and uploaded all figures (Figures 1–6). Figure 1 was regenerated in TIF format, while Figures 2–6 were regenerated as EPS vector files to achieve higher resolution, ensuring that legends, axis labels, and numerical values are clearly legible.

*4.Missing Relevant Graph-Based Literature*

**RE:** We thank the reviewer for this valuable suggestion. In the revised manuscript, we have incorporated the recommended works into the Related Work section. Specifically, before introducing Int-GNN, we now discuss the Graph Time Neural Network (GTN) and the self-evolutionary Graph Isomorphic Network (GIN). These studies provide valuable perspectives on how graph-based attention mechanisms and self-evolutionary strategies can effectively address complex sequential dependencies and cross-scenario modeling in safety-critical engineering systems. By placing them before Int-GNN, we highlight that similar challenges—such as capturing long-term dependencies and ensuring robust generalization—also arise in other domains. This contextualization provides new insights that inspired our work and underscores the novelty of MSGNN in addressing analogous issues within session-based recommendation.For revisions made to the paper, please refer to the paragraphs marked in the revised draft, specifically the relevant sections. [The newly added content in the draft revision is highlighted in yellow on page 4 of the revised version:](D:\\论文-PPT\\论文\\plos one\\ESP\\Marked-up copy of the manuscript.pdf" \l "page=4)

Beyond recommender systems, graph-based models have also been applied in safety-critical dynamic system prediction tasks. For instance, Peng et al[37]. proposed the Graph Time Neural Network (GTN), which integrates graph attention mechanisms with multi-scale time series analysis and auxiliary learning to enhance long-term prediction in train–bridge coupled systems. Similarly, Zhang et al[38]. introduced a self-evolutionary Graph Isomorphic Network (GIN) for multi-scenario safety assessment of railway bridges, demonstrating robust performance across both known and unseen scenarios. These studies provide new perspectives and inspire our work by showing how graph-based attention and self-evolutionary strategies can effectively address complex sequential dependencies and cross-scenario modeling.In the context of session-based recommendation, Int-GNN[39] represents an attempt to incorporate frequency-based signals to model user intent within sessions, achieving competitive performance. However, consistent with the challenges identified in other domains such as dynamic system prediction and cross-scenario safety assessment, Int-GNN still suffers from key limitations: it focuses only on single-session modeling, overlooks cross-session intent dynamics, and lacks effective strategies for handling data sparsity—one of the most common challenges in SBR. Building on the new perspectives provided by recent graph-based advances in other fields and addressing the specific shortcomings of Int-GNN, our study proposes a more robust and generalizable session-based recommendation model.

[37] Peng X, Zhang P, Huang Z, Xie X, Liu Z, Chen Y, et al. Graph-based attention model for predictive analysis in train-bridge systems Applied Soft Computing, 2025: 113360.

<https://www.sciencedirect.com/science/article/abs/pii/S1568494625006714>

[38] Zhang P, Zhao H, Shao Z, Xie X, Hu H, Zeng Y, et al. Enhanced multi-scenario running safety assessment of railway bridges based on graph neural networks with self-evolutionary capability Engineering Structures, 2024;319:118785.

<https://www.sciencedirect.com/science/article/abs/pii/S0141029624013476>

**Reviewer :2**

*1.The abstract states that a new model of GCACL-Rec is proposed, but the title, body and conclusion refer to “MSGNN” several times (e.g. “MSGNN: A Study on...”). , please explain briefly. In addition, please explain whether the first appearance of GCACL-Rec is defined and how the most central innovation of the model is reflected.*

**RE:** We sincerely thank the reviewer for pointing out this inconsistency. You are absolutely right — our initial title may have caused confusion by highlighting “MSGNN,” which is actually a core component of our proposed model, GCACL-Rec, rather than the model itself. The full model GCACL-Rec includes additional key components such as multi-perspective contrastive adversarial learning and hybrid prediction modules. To address this, we have made the following revisions:

(1) Revised the title of the manuscript to accurately reflect the proposed model name, GCACL-Rec, and ensure consistency with the abstract, main text, and conclusion.

GCACL-Rec: A Study on Conversational Recommendation via Global Context-Aware and Multi-View Contrastive Adversarial Joint Learning

(2) In the methodology section, we have clarified that GCACL-Rec is the overall model architecture, and MSGNN is one of its core modules responsible for multi-scale global item transition modeling.

To address the above challenges, we propose GCACL-Rec (Global Context-Aware Contrastive Learning for Recommendation), a novel session-based recommendation model that integrates multiple components for cross-session modeling, contrastive learning, and decision fusion. At its core, GCACL-Rec includes a global-level Multi-Scale Graph Neural Network (MSGNN) to capture complex item transitions across sessions and a local-level Position-aware Graph Neural Network (P-GNN) to model sequential dependencies within individual sessions. MSGNN incorporates external hypernodes that aggregate semantically related sessions, enabling cross-session message passing through node–hypernode interactions. To further enhance this process, a relative multi-head attention mechanism is applied to capture position-sensitive dependencies across nodes.

*2.Are hyperparameter settings (e.g., number of super-nodes, model depth) determined through systematic tuning experiments? Are parameter sensitivity analyses (e.g., learning rate, number of layers vs. performance curve) provided to support the rationality of the current choices?*

**RE:** We sincerely thank the reviewer for raising this important question. We recognize that conducting systematic hyperparameter tuning and providing sensitivity analyses are critical for ensuring the robustness and rationality of the model. In response to this comment, we have performed systematic experiments to determine the best hyperparameter settings for our model, and we have included sensitivity analyses to further support the rationale behind our choices.

For the core hyperparameters, including the number of hypernodes and model depth, we conducted grid search experiments on the validation set to identify the optimal values. The number of hypernodes was tested with values ranging from 2 to 12. Through these experiments, we found that 4 hypernodes achieved the best performance in capturing global context while avoiding overfitting. Similarly, we experimented with model depths ranging from 1 to 5 layers. The 3-layer configuration demonstrated the best trade-off between performance and model complexity, effectively capturing item transitions across sessions without overfitting. You can find the detailed hyperparameter tuning results in Results and the corresponding Figures 4 and 5 where we show the performance curves for different values of the number of hypernodes and model depth.

In addition to hyperparameter tuning, we performed parameter sensitivity analyses to evaluate the impact of critical training parameters on model performance. We tested multiple learning rates (e.g., 0.001, 0.0015, 0.002, 0.025) and found that a learning rate of 0.0015 yielded the best performance, as demonstrated in Figure 6. Additionally, we performed a grid search on the contrast loss weight (μ) within the range of 0.1 to 0.25, with 0.1 being the optimal value. This value achieves the best balance between contributing to contrast learning and avoiding over-penalizing the model's ability to fit the data. The specific experimental content can be found in Figure 6 and Table 3 in the Sensitivity Analysis section of RQ6 in the revised draft. [The specific modifications are highlighted in yellow on page 20 of the revised draft:](D:\\论文-PPT\\论文\\plos one\\ESP\\Marked-up copy of the manuscript.pdf" \l "page=20)

RQ6-Sensitivity Analysis

To validate the rationality of our chosen hyperparameters, we first conducted a sensitivity experiment on the learning rate. On the RetailRocket dataset, we tested four values of learning rate (lr) at 0.001, 0.0015, 0.002, and 0.025, and plotted the curves of Recall@20 and MRR@20 over epochs (in Fig 6). The results show that a smaller learning rate (0.001) ensures stable convergence but converges more slowly overall; moderate learning rates (0.0015 and 0.002) lead to rapid performance gains in the early epochs and eventually achieve higher Recall and MRR, with lr = 0.0015 performing best, reaching about 58.98 in Recall@20 and 32.52 in MRR@20. In contrast, a substantial learning rate (0.025) improves quickly in the early stage but exhibits significant fluctuations later, indicating instability in training. Thus, lr = 0.0015 strikes a good balance among convergence speed, final performance, and stability, making it the preferable choice.

We further performed sensitivity analysis on the contrastive loss parameter μ, with results shown in Table 3. When μ = 0.10 and μ = 0.15, the model achieved superior and stable performance, with μ = 0.10 performing best (Recall@20 = 55.48, MRR@20 = 19.81). In contrast, when μ = 0.20 and μ = 0.25, performance dropped noticeably, with Recall@20 falling to around 53.74. This suggests that an overly large μ assigns excessive weight to the contrastive loss, thereby weakening the optimization of the primary recommendation task. Considering both performance and stability, μ = 0.10 is the optimal setting. Overall, the final parameter configuration (lr = 0.0015, μ = 0.10) was determined through extensive empirical validation and ensures consistent and strong performance on both Recall and MRR, thus supporting the soundness of our parameter choices.

*3.Is the “increase in computational complexity” mentioned in the model limitations section quantified by a time complexity analysis? Are potential optimization directions discussed (e.g., model compression, distributed training)?*

**RE:** We sincerely thank the reviewer for this question. We fully acknowledge that computational complexity is a crucial consideration for real-world deployment, and we appreciate the opportunity to clarify and improve our work on this point.

In the current version of the manuscript, while we mention a possible increase in computational complexity due to the integration of multiple modules (e.g., MSGNN and P-GNN), we did not provide a formal time complexity analysis to quantify this issue. We realize that simply stating the existence of additional computational cost without a quantitative discussion may be insufficient for evaluating the scalability of our model. To address this, we will include a more detailed analysis of time complexity in future work. This will involve quantifying the computational complexity of the key components, particularly MSGNN and P-GNN, and comparing them with other state-of-the-art methods to better understand the trade-offs in efficiency. We also acknowledge that potential optimization directions, such as model compression and distributed training, are not explicitly discussed in the current manuscript. These are indeed valuable strategies for improving training and inference efficiency, especially when dealing with large-scale datasets or resource-constrained environments. In future work, we plan to explore several optimization techniques: Techniques such as quantization, pruning, and knowledge distillation will be considered to reduce model size and computational cost while preserving performance. As the proposed model integrates multiple components and is trained on large datasets, we will investigate multi-GPU or distributed training frameworks to accelerate training and enhance scalability. [The specific modifications are highlighted in yellow on page 21 of the revised draft:](D:\\论文-PPT\\论文\\plos one\\ESP\\Marked-up copy of the manuscript.pdf" \l "page=21)

For future work, we plan to mitigate computational complexity by applying model compression techniques such as pruning and quantization, reducing model size and improving inference speed without sacrificing performance. We will also explore distributed training with multi-GPU setups and cloud platforms to accelerate training and scale to larger datasets. These efforts are essential for enhancing the efficiency and real-world applicability of the proposed model. In summary, GCACL-Rec provides an effective solution for cross-session fusion, high-order behavior modeling, and self-supervised learning, offering a strong foundation for personalized recommendations in complex scenarios.

*4. The manuscript reports overall performance but does not analyze error cases in depth. Can you provide a qualitative analysis of high-frequency error patterns (e.g., mistakenly pushing associated items)?*

**RE:** We thank the reviewer for this constructive suggestion. We fully agree that analyzing error cases can provide deeper insights into the strengths and limitations of different models. In the revised manuscript, we have expanded the analysis in the Results, RQ1 section. Specifically, we now provide a more detailed discussion of the baseline methods by describing not only their modeling strategies but also the typical errors they are prone to. [The specific amendments on pages 16 and 17 of the draft revision are highlighted in purple:](D:\\论文-PPT\\论文\\plos one\\ESP\\Marked-up copy of the manuscript.pdf" \l "page=16)

To overcome the limitations of traditional methods, recent research has shifted toward graph neural network (GNN) based modeling. SR-GNN employs gated graph neural networks to capture pairwise item transitions within sessions, but it often recommends semantically related yet incorrect items (e.g., predicting a charger instead of a phone case) due to the lack of higher-order dependency modeling. NISER normalizes item and session embeddings to reduce popularity bias, but under sparse sessions, it struggles to retain fine-grained sequential cues, leading to context-mismatched predictions. GCE-GNN extends graph modeling across sessions through session-level and global graphs, but it tends to overemphasize global co-occurrence while neglecting intra-session transitions. S2-DHCN leverages hypergraphs for high-order relations, yet without explicit transition direction, it fails to recover next-item flow, and under sparsity, high-order co-occurrence amplifies noise. COTREC enhances robustness with dual graph views and contrastive co-training, but struggles with nonlinear or complex user intents beyond its consistency objective. Int-GNN integrates item frequency and re-interaction intervals for intent inference, but becomes unstable in short or anonymous sessions where global statistics are insufficient.

Compared with the above baseline methods, the proposed GCACL-Rec demonstrates consistent advantages across all datasets and metrics. On average, GCACL-Rec outperforms the best baseline by 1.48\% on Diginetica, 5.92\% on Tmall, and 2.74\% on RetailRocket. This gain mainly comes from two components: (i) a global, multi-scale graph with hypernodes that captures high-order item transitions within and across sessions, together with a relative multi-head attention mechanism that enhances cross-session propagation by modeling position-sensitive dependencies, thereby alleviating errors such as “associated but non-target” predictions; and (ii) multi-view adversarial contrastive learning coupled with a neural decision forest, which improves robustness under sparsity, mitigates popularity bias, and models nonlinear intent patterns more effectively. Overall, GCACL-Rec addresses the key limitations observed in prior work—pairwise-only modeling, popularity bias under sparsity, missing transition direction, and instability in short or anonymous sessions—while achieving state-of-the-art results across benchmarks.

Following this discussion, we explicitly connect these shortcomings to the improvements achieved by our proposed GCACL-Rec. This addition makes clear how our model addresses common error patterns—by capturing both pairwise and high-order transitions, mitigating popularity bias, and modeling nonlinear intent dynamics.

5. *Is the “stable performance in sparse and cold start scenarios” mentioned in the conclusion based on sufficient experimental evidence? Are comparative performance data for specific scenarios provided?*

**RE:** We thank the reviewer for this valuable comment. Regarding the cold-start scenario, we have provided additional parameter sensitivity analyses (see Fig. 6), where the performance of our model was evaluated under different learning rates. The results clearly show that even in the very early training epochs (i.e., with limited training data, which resembles a cold-start situation), GCACL-Rec quickly converges to competitive performance. In particular, with a learning rate of 0.0015, the model reaches near-optimal Recall@20 and MRR@20 within the first 2–3 epochs, demonstrating stable and reliable recommendation quality during the cold-start phase.

With respect to sparse data scenarios, our overall experimental results on the Tmall and RetailRocket datasets (both of which are relatively sparser compared to Diginetica) show consistent improvements over the baselines. The gains are more pronounced on these datasets, indicating that GCACL-Rec is effective in alleviating the data sparsity problem. These improvements, together with the fast convergence observed in the sensitivity experiments, provide sufficient empirical evidence to support the statement in our conclusion that GCACL-Rec exhibits stable performance in both sparse and cold-start scenarios.

*6. Table 2 shows that its performance metrics behave erratically on different datasets. Please explain its rationale.*

**RE:** We thank the reviewer for this comment. We acknowledge that the performance gains of GCACL-Rec are not uniform across datasets in Table 2. This variation can be explained by the inherent differences in dataset characteristics, which influence how effectively our model’s components contribute to the recommendation task. Specifically, Diginetica and RetailRocket contains relatively short average sessions (Table 1). Since our model leverages high-order dependencies through the multi-scale graph and hypernodes, shorter sessions limit the benefits of this structural modeling. This explains the relatively modest improvements on Diginetica. Besides, Tmall and RetailRocket are comparatively more sparse, with larger item vocabularies and fewer interactions per item. Under such conditions, GCACL-Rec’s global structural modeling and adversarial contrastive learning help mitigate sparsity and popularity bias, leading to more substantial gains, and each dataset reflects different domains (e.g., e-commerce logs with varying item types and user behaviors). These domain-specific characteristics affect how well sequential dependencies and intent signals can be captured, contributing to the observed performance variations.

We have revised the “Results/RQ1” section to clearly highlight these factors related to the dataset. Therefore, the research results are not unstable, but consistent with the expected impact of dataset sparsity, session duration, and domain heterogeneity on session-based recommendation systems. [The specific amendments on pages 17 of the draft revision are highlighted in green:](D:\\论文-PPT\\论文\\plos one\\ESP\\Marked-up copy of the manuscript.pdf" \l "page=17)

Compared with the above baseline methods, the proposed GCACL-Rec demonstrates consistent advantages across all datasets and metrics. On average, GCACL-Rec outperforms the best baseline by 1.48\% on Diginetica, 5.92\% on Tmall, and 2.74\% on RetailRocket. This gain mainly comes from two components: (i) a global, multi-scale graph with hypernodes that captures high-order item transitions within and across sessions, together with a relative multi-head attention mechanism that enhances cross-session propagation by modeling position-sensitive dependencies, thereby alleviating errors such as “associated but non-target” predictions; and (ii) multi-view adversarial contrastive learning coupled with a neural decision forest, which improves robustness under sparsity, mitigates popularity bias, and models nonlinear intent patterns more effectively. The performance differences across datasets can be attributed to their distinct characteristics (Table 1). Diginetica has shorter sessions, which reduces the benefit of high-order structural modeling, leading to relatively smaller improvements. By contrast, Tmall and RetailRocket are more sparse and contain larger item vocabularies, where the proposed global multi-scale graph and adversarial contrastive learning are more effective in alleviating sparsity and popularity bias, resulting in larger relative gains. Overall, GCACL-Rec addresses the key limitations observed in prior work—pairwise-only modeling, popularity bias under sparsity, missing transition direction, and instability in short or anonymous sessions—while achieving state-of-the-art results across benchmarks.

*7.(1)Does the baseline model selection cover different technical routes (e.g., RNN, GNN, Transformer-based approaches)?*

**RE:** Thank you very much for your valuable suggestions. We agree that the baseline model should cover different technical approaches, especially Transformer-based methods, as this would make the comparative experiments in the paper more comprehensive and persuasive. We fully agree with your perspective. In the initial submission, we primarily compared RNN-based models (such as GRU4Rec), Attention-based models (such as NARM, STAMP), and GNN-based models (such as SR-GNN, GCE-GNN). As you pointed out, there was a lack of comparison with classic Transformer-based models.

In response to your feedback and to ensure fairness in the comparison, we have now supplemented the experimental results with two of the most widely recognized Transformer baseline models in the field of conversation recommendation: SASRec [24] and BERT4Rec [53]. These results are sourced from the literature [54], which were conducted under unified data preprocessing and segmentation standards. This ensures that all models are compared under identical experimental conditions, thereby avoiding performance biases caused by inconsistent data processing. We have added the results of SASRec and BERT4Rec to Table 2 (main experimental results table) in the revised draft (as shown below). The results demonstrate that our proposed GCACL-Rec model consistently outperforms these powerful Transformer baselines across nearly all metrics, further validating the effectiveness and advanced nature of our model. Once again, we sincerely thank you for your rigorous and professional review, which has significantly enhanced the quality of our paper. [The specific modifications are highlighted in orange in the revised draft:](D:\\论文-PPT\\论文\\plos one\\ESP\\Marked-up copy of the manuscript.pdf)

[24] Kang W C, McAuley J. Self-attentive sequential recommendation. 2018 IEEE

international conference on data mining (ICDM). 2018. p. 197-206.

<https://doi.org/10.1109/ICDM.2018.00035>

[53] Sun F, Liu J, Wu J, Pei C, Lin X, Ou W, et al. BERT4Rec: Sequential recommendation with bidirectional encoder representations from transformer In: Proceedings of the 28th ACM international conference on information and knowledge management. 2019. p. 1441-1450.

<https://dl.acm.org/doi/abs/10.1145/3357384.3357895>

[54] Wan Z, Liu X, Wang B, Qiu J, Li B, Guo T, et al. Spatio-temporal contrastive

learning-enhanced GNNs for session-based recommendation ACM Transactions

on Information Systems, 2023;42(2):1-26.<https://dl.acm.org/doi/abs/10.1145/3626091>

Baseline models

To evaluate the model’s performance, we comprehensively evaluate the performance of GCACL-Rec with 14 baselines from 5 different types:

Methods based on frequency and matrix decomposition

• POP: It employs item frequency statistics to recommend the top-N most popular

items from the training set.

• FPMC [6]: It employs matrix factorization combined with a Markov chain to

capture short-term item transitions.

RNN modeling methods

• GRU4Rec [10]: It is an RNN-based model that uses a Gated Recurrent Unit

(GRU) to model user sequences.

• NARM [4]: It employs an RNN encoder to capture sequential context, followed by

an attention mechanism to focus on dominant user intent.

Attention mechanism methods

• STAMP [5]: It employs attention layers to replace all RNN encoders in previous

work by fully relying on the self-attention of the last item in the current session to

capture the user’s short-term interest.

Transformer-based methods

• SASRec [24]: It employs stacked self-attention layers with positional encoding to

capture sequential dependencies, followed by unidirectional modeling to predict

the next item in the sequence.

• BERT4Rec [53]: It employs bidirectional Transformer encoders with a masked

item prediction objective, followed by contextualized embeddings to capture both

left and right dependencies for sequential recommendation.

Graph neural network methods

• SR-GNN [15]: It employs a gated GNN layer to obtain item embeddings, followed

by a self-attention of the last item as STAMP [5] does to compute the session-level

embeddings for session-based recommendation.

• NISER [32]: It employs GNN-based item modeling, followed by L2 regularization,

to mitigate the effects of long-tail distribution.

• LESSR [51]: It employs a GRU-enhanced graph structure to learn item

representations, followed by session-level aggregation.

• GCE-GNN [37]: It employs global and session graphs jointly to learn item

embeddings, followed by a fusion layer to enhance the cross-session context.

• S2-DHCN [33]: It employs a hypergraph and line graph to model high-order

relations, followed by contrastive learning to optimize representations.

• COTREC [52]: It employs dual graph encoders trained with self-supervised

signals, followed by fusion to model intra- and inter-session structures.

• Int-GNN [38]: It employs frequency, re-interaction gaps, and user preferences to

guide intent modeling, followed by GNN-based feature extraction.

Datasets Diginetica	Tmall	RetailRocket

Metrics P@20 MRR@20 P@10 MRR@10 P@20 MRR@20 P@10 MRR@10 P@20 MRR@20 P@10 MRR@10

POP 1.18 0.28 0.76 0.26 2.00 0.90 1.67 0.88 1.12 0.30 0.61 0.27

FPMC 22.14 6.66 15.43 6.20 16.06 7.32 13.10 7.12 32.37 13.82 25.99 13.38

GRU4Rec 29.45 8.33 17.93 7.33 10.93 5.89 9.47 5.78 44.01 23.67 38.35 23.27

NARM 49.70 16.17 35.44 15.13 23.30 10.70 19.17 10.42 50.22 24.59 42.07 24.88

STAMP 45.64 14.32 33.98 14.26 26.47 13.36 22.63 13.12 50.96 25.17 42.95 24.61

SASRec 48.78 17.22 35.84 14.55 27.72 12.11 21.91 11.25 45.85 23.39 37.55 22.12

BERT4Rec 50.12 17.16 36.78 15.61 28.12 12.85 22.38 11.58 46.72 25.52 38.92 23.44

SR-GNN 50.73 17.59 36.86 15.52 27.57 13.72 23.41 13.45 50.32 26.57 43.21 26.07

NISER 53.39 18.72 40.20 17.82 33.79 16.67 28.46 16.38 54.90 29.89 47.69 29.38

LESSR 51.71 18.15 36.16 15.64 27.88 12.08 22.68 11.68 53.05 28.01 45.76 27.51

GCE-GNN 54.22 19.04 41.16 18.15 33.42 15.24 28.01 15.08 50.60 25.39 43.53 24.89

S2-DHCN 53.66 18.51 40.21 17.59 31.42 15.05 26.22 14.60 53.66 27.30 46.15 26.85

COTREC 54.18 19.07 41.88 18.16 36.35 18.04 30.62 17.65 56.17 29.97 48.61 29.46

Int-GNN 55.16 19.46 41.84 18.53 40.77 18.20 34.28 17.74 58.02 31.48 50.41 30.94

GCACL-Rec 55.48 19.81 42.33 18.89 42.30 19.47 35.79 19.02 58.98 32.52 51.19 32.00

Imp 0.65% 1.91% 1.31% 2.05% 4.16% 7.69% 4.77% 7.09% 1.79% 3.67% 1.71% 3.80%

Note: Results for SASRec and BERT4Rec are sourced from [54] to ensure a fair comparison under consistent data preprocessing and splitting standards. The best-performing results are labeled in bold, and the relative performance improvement of our model compared to the best-performing baseline is labeled with ‘Imp’.

Compared with neural network-based session recommendation methods, traditional models show apparent limitations in modeling sequential dependencies. GRU4Rec was the first to apply gated recurrent units (GRU) to user behavior sequences, initiating the use of deep learning in session-based recommendation. Later methods, such as NARM and STAMP, introduced attention mechanisms to focus on key behaviors, significantly improving performance. However, they mainly capture explicit interactions while ignoring the implicit relationships between items. Subsequent Transformer-based approaches, such as SASRec and BERT4Rec, further advanced sequential modeling by leveraging self-attention to capture long-range dependencies. While SASRec employs an unidirectional encoder to predict the next item, BERT4Rec adopts bidirectional context through a masked prediction objective. However, both methods still face challenges in modeling higher-order item relations and session-level intent.

Thank you once again for your rigorous and professional review. Your comments have greatly improved the quality of our paper.

*(2) In addition, it is necessary to cite session recommendations of recent reviews or GNN comparative learning frontier work to highlight the work of this study.*

**RE:** Thank you for the valuable suggestion. In the revised manuscript, we have incorporated recent advances in both session-based recommendation and graph-based contrastive learning to better position our work within the current research landscape. Specifically, we added LightGCL [44] and SimGCL [45], two recent GNN-based contrastive learning methods that streamline the framework and improve efficiency. For session-based recommendation, we included Self-Contrastive Learning (SCL) [48] and RecDCL [49], which represent the latest developments addressing challenges such as user anonymity, noisy interactions, and representation robustness. Additionally, we referenced knowledge-grounded dialogue (KGD) [46] and TimesURL [47] from other domains (NLP and time series), both of which demonstrate how contrastive learning has been extended with task-specific designs. These updates make our Related Work section more comprehensive and highlight how our proposed MPACL framework goes beyond predefined augmentations by incorporating adversarial training and multi-perspective alignment for more robust intent modeling. [The specific modifications are highlighted in blue on page 4 of the revised draft:](D:\\论文-PPT\\论文\\plos one\\ESP\\Marked-up copy of the manuscript.pdf" \l "page=4)

Contrastive Learning (CL) is a self-supervised paradigm that extracts supervisory signals from unlabeled data through pretext tasks. Its core idea is to learn robust representations by maximizing agreement between different augmented views of the same instance (positives) while minimizing similarity with views of other instances (negatives), typically optimized via contrastive loss. CL first achieved breakthroughs in computer vision with models such as SimCLR [40] and MoCo [41], and has since been extended to natural language processing [42] and audio processing [43].

Recently, CL has gained traction in recommender systems for alleviating data sparsity and improving representation learning. LightGCL [44] employs singular value decomposition (SVD) for global alignment, while SimGCL [45] demonstrates that injecting uniform noise into embeddings achieves strong performance at lower cost. Building on these foundations, the principles of CL—view-invariant representation and hardness-aware negative sampling—have been adapted to domain-specific challenges. For example, in knowledge-grounded dialogue (KGD) [46], entity-aware CL improves robustness by constructing positives with irrelevant noise and negatives with relevant noise, while in time series, TimesURL [47] leverages frequency-temporal augmentations and dual negative sampling to learn generalizable representations.

In session-based recommendation (SBR), CL has been applied to handle user anonymity and noisy interactions. SCL [48] simplifies CL by directly optimizing alignment and uniformity without complex augmentations, improving interpretability and efficiency. RecDCL [49] further introduces a dual framework with batch-wise and feature-wise objectives for enhanced robustness. However, most methods still rely on fixed augmentations and overlook adversarial and multi-perspective alignment. To address this, we propose Multi-Perspective Adversarial Contrastive Learning (MPACL), which builds tailored session views and employs adversarial training to generate challenging negatives, thereby distilling true user intent from noise and improving both robustness and discriminative power of session representations.

[44] Cai X, Huang C, Xia L, Ren X. LightGCL: Simple yet effective graph contrastive learning for recommendation. arXiv preprint 2023.

<https://doi.org/10.48550/arXiv.2302.08191>

[45] Yu J, Yin H, Xia X, Chen T, Cui L, Nguyen Q V H. Are graph augmentations

necessary? simple graph contrastive learning for recommendation. In: Proceedings of the 45th international ACM SIGIR conference on research and development in information retrieval. 2022. p. 1294-1303. https://doi.org/10.1145/3477495.3531937

[46] Wang J, Qu J, Wang K, Li Z, Hua W, Li X, et al. Improving the robustness of knowledge-grounded dialogue via contrastive learning. In: Proceedings of the AAAI Conference on Artificial Intelligence. 2024;38(17):19135-19143.

https://ojs.aaai.org/index.php/AAAI/article/view/29881

[47] Liu J, Chen S. Timesurl: Self-supervised contrastive learning for universal time series representation learning. In: Proceedings of the AAAI conference on artificial intelligence. 2024;38(12):13918-13926.

https://ojs.aaai.org/index.php/AAAI/article/view/29881

[48] Shi Z, Wang X, Lipani A. Self contrastive learning for session-based recommendation. European Conference on Information Retrieval. Cham: Springer Nature Switzerland. 2024. p. 3-20.

[49] Zhang D, Geng Y, Gong W, Qi Z, Chen Z, Tang X, et al. Recdcl: Dual contrastive learning for recommendation. In: Proceedings of the ACM Web Conference 2024. 2024. p. 3655-3666.

https://dl.acm.org/doi/abs/10.1145/3589334.3645533
